# Changes in plant biodiversity facets of rocky outcrops and their surrounding rangelands across precipitation and soil gradients

**DOI:** 10.1038/s41598-022-13123-2

**Published:** 2022-05-30

**Authors:** Fahime Rafiee, Hamid Ejtehadi, Mohammad Farzam, Habib Zare, Maral Bashirzadeh

**Affiliations:** 1 Quantitative Plant Ecology and Biodiversity Research Lab, Department of Biology, Faculty of Science, Ferdowsi University of Mashhad, Mashhad, Iran; 2 Department of Range and Watershed Management, Faculty of Natural Resources and Environment, Ferdowsi University of Mashhad, Mashhad, Iran; 3 Botanical Garden of Nowshahr, Research Institute of Forests and Rangelands, Agricultural Research, Education and Extension Organization, AREEO, Tehran, Iran

**Keywords:** Biodiversity, Community ecology, Ecology

## Abstract

Climate and soil factors induce substantial controls over plant biodiversity in stressful ecosystems. Despite of some studies on plant biodiversity in extreme ecosystems including rocky outcrops, simultaneous effects of climate and soil factors have rarely been studied on different facets of biodiversity including taxonomic and functional diversity in these ecosystems. In addition, we know little about plant biodiversity variations in such extreme ecosystems compared to natural environments. It seems that environmental factors acting in different spatial scales specifically influence some facets of plant biodiversity. Therefore, we studied changes in taxonomic and functional diversity along precipitation and soil gradients in both landscapes (i) rocky outcrops and (ii) their nearby rangeland sites in northeast of Iran. In this regard, we considered six sites across precipitation and soil gradients in each landscape, and established 90 1m^2^ quadrates in them (i.e. 15 quadrats in each site; 15 × 6 = 90 in each landscape). Then, taxonomic and functional diversity were measured using RaoQ index, FDis and CWM indices. Finally, we assessed impacts of precipitation and soil factors on biodiversity indices in both landscapes by performing regression models and variation partitioning procedure. The patterns of taxonomic diversity similarly showed nonlinear changes along the precipitation and soil factors in both landscapes (i.e. outcrop and rangeland). However, we found a more negative and significant trends of variation in functional diversity indices (except for CWMSLA) across precipitation and soil factors in outcrops than their surrounding rangelands. Variations of plant biodiversity were more explained by precipitation factors in surrounding rangelands, whereas soil factors including organic carbon had more consistent and significant effects on plant biodiversity in outcrops. Therefore, our results represent important impacts of soil factors in structuring plant biodiversity facets in stressful ecosystems. While, environmental factors acting in regional and broad scales such as precipitation generally shape vegetation and plant biodiversity patterns in natural ecosystems. We can conclude that rocky outcrops provide suitable microenvironments to present plant species with similar yields that are less able to be present in rangeland ecosystems.

## Introduction

Rocky outcrops and cliffs have been identified as one of the most important micro-habitats in stressful ecosystems^1^. These micro-habitats are known by inaccessible topography to humans and livestock, high environmental changes, and low levels of biotic interactions, which provided safe sites for the native, relic, and endemic species^2–4^. On the other hand, they are characterized by shallow soils, low water and nutrients, low night temperatures, high sunlight, and strong winds^3,5,6^. These fragmented and isolated natural habitats are characterized by spatial and ecological segregation that hinder dispersal and migration due to environmental and geographical conditions^7–9^. Despite the importance of these ecosystems, biodiversity studies have been conducted less in them and need further study^10–14^. Therefore, it is necessary to examine the impacts of different environmental factors and rocky outcrops on biodiversity to understand better the factors influencing biodiversity patterns.

Outcrops are recognized as biodiversity hotspots for plants with specific adaptive traits, such as tolerance to environmental stresses^15^. Certain studies have also shown a degree of floristic similarity between the outcrops^16–19^ with a low degree of genetic exchange rates between different rock outcrop populations^20–24^. Such features of outcrops have led to some differences in biodiversity between these specific ecosystems with their surrounding environments, in which environmental factors indirectly handle such differences^25–28^. However, relative importance of environmental factors acting in different spatial scales such as climatic factors in regional scale compared to soil factors in fine scales on shaping such differences between outcrops and their surrounding environments is unknown^29^.

Understanding the processes shaping the biodiversity in outcrops is necessary due to shed light in relative importance the environmental factors on biodiversity of outcrops^30^. Biodiversity in outcrops may be associated to variation in some environmental factors such as soil depth, soil fertility, topographic and climatic gradients^31,32^. Various studies have shown the effects of precipitation and soil fertility on taxonomic and functional diversity in some outcrops located in northeastern Iran and Spain^25,29,33–39^. Precipitation is much more pronounced in arid and semi-arid ecosystems than other climatic factors, with an important impact on shaping the biodiversity at regional spatial scales^35,40^. Whereas, soil factors indicated important effects on structuring biodiversity facets at fine and local spatial scales^41–45^.

Most of studies on the flora and biodiversity of rocks and outcrop communities have focused on taxonomic diversity^3,46–51^. Biodiversity is not just the diversity of species present in a community, but a multiple structure and concept calculated and interpreted through a wide range of genes, species, and functional traits in the ecosystem^52,53^. Taxonomic diversity may give us minor information about all dimensions of biodiversity. For example, plant diversity can have precisely the same taxonomic diversity but very different levels of functional diversity resulting in very different levels of biodiversity^53,54^. Furthermore, approaches based on functional traits have come out as a promising way to understand plant ecological strategies, plant interactions, and their linkages to ecosystem functioning^55–59^. Therefore, it is necessary to measure plant functional diversity simultaneous with taxonomic diversity for a mechanistic understanding the impacts of different environmental factors on structuring the biodiversity in outcrops and their surrounding environments^60,61^.

This study was conducted on limestone outcrops in northern Iran, along a cross transaction of Alborz Mountain Ranges from Shahroud (dry steppe rangeland) to Gorgan (temperate forest). Various geological and topographical situations, huge climatic contrasts, and a long history of evolution in this region make its rocky outcrops and surrounding rangelands as interesting and challenging environments for study^62–68^. However, studies on biodiversity of outcrops and their surrounding rangelands in Iran are limited to measuring taxonomic diversity^69–73^. In addition, relative importance of environmental factors on structuring of biodiversity facets and degree of correlation between outcrops and their surrounding rangelands with respect to biodiversity facets are not recognized so far. Therefore, we investigated the effects of precipitation and soil factors on taxonomic and functional diversity in the outcrops and their surrounding rangelands. In addition, we tested relative importance of these environmental factors to explain variations in taxonomic and functional diversity, and addressed following questions: (1) how the taxonomic and functional diversity vary across precipitation and soil factors with respect to ecosystem considered (i.e. outcrops vs surrounding rangelands)? (2) Do precipitation and soil factors exhibit significant effects on structuring taxonomic and functional diversity, and if so, by how much?

## Results

### Plant taxonomic and functional diversity across precipitation and soil factors

**Figure 1:**
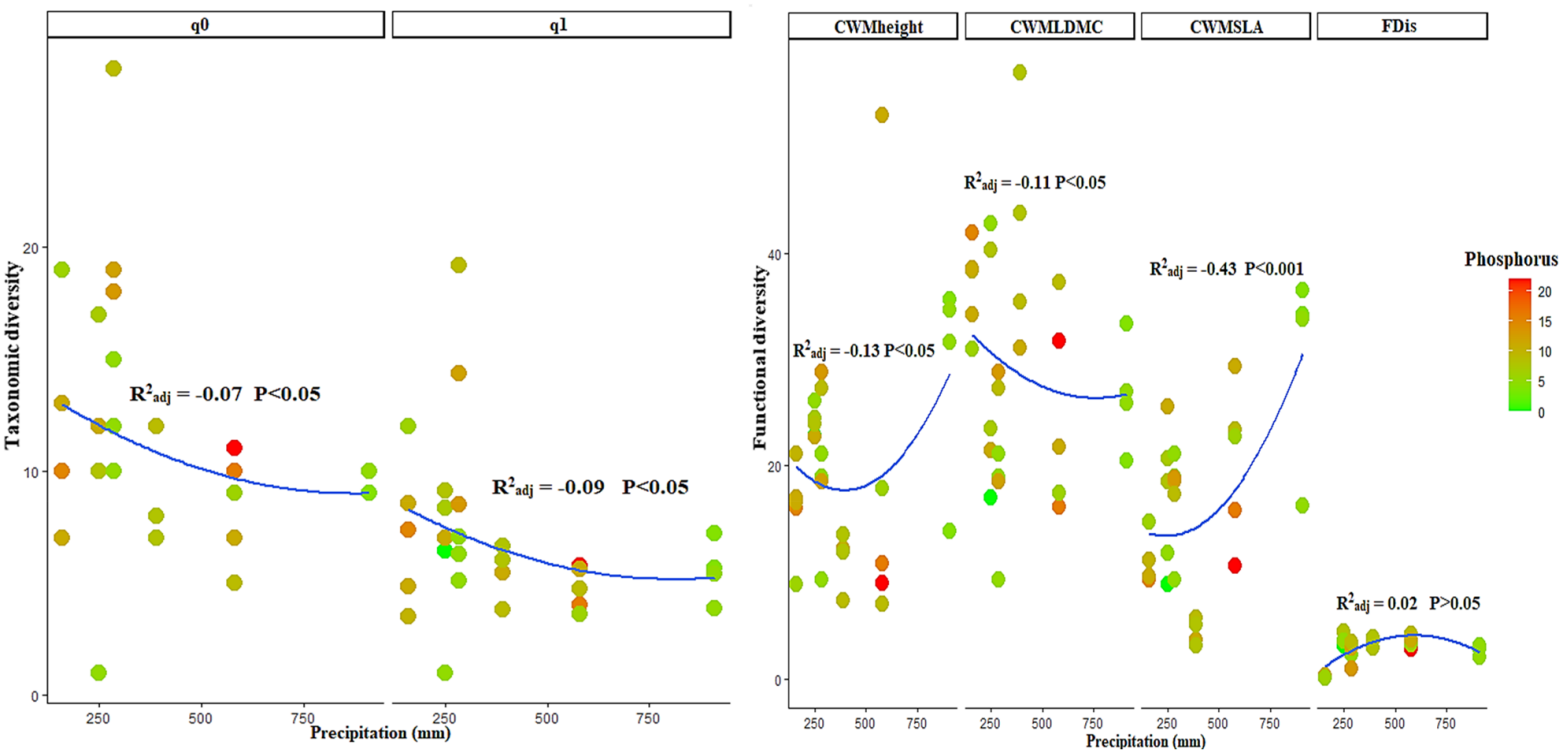
Variation in taxonomic (q_0_ and q_1_ in left panels) and functional (CWMheight, CWMLDMC, CWMSLA and FDis in right panels) diversity in rangeland sites across precipitation and phosphorus (P) gradients.

**Figure 2:**
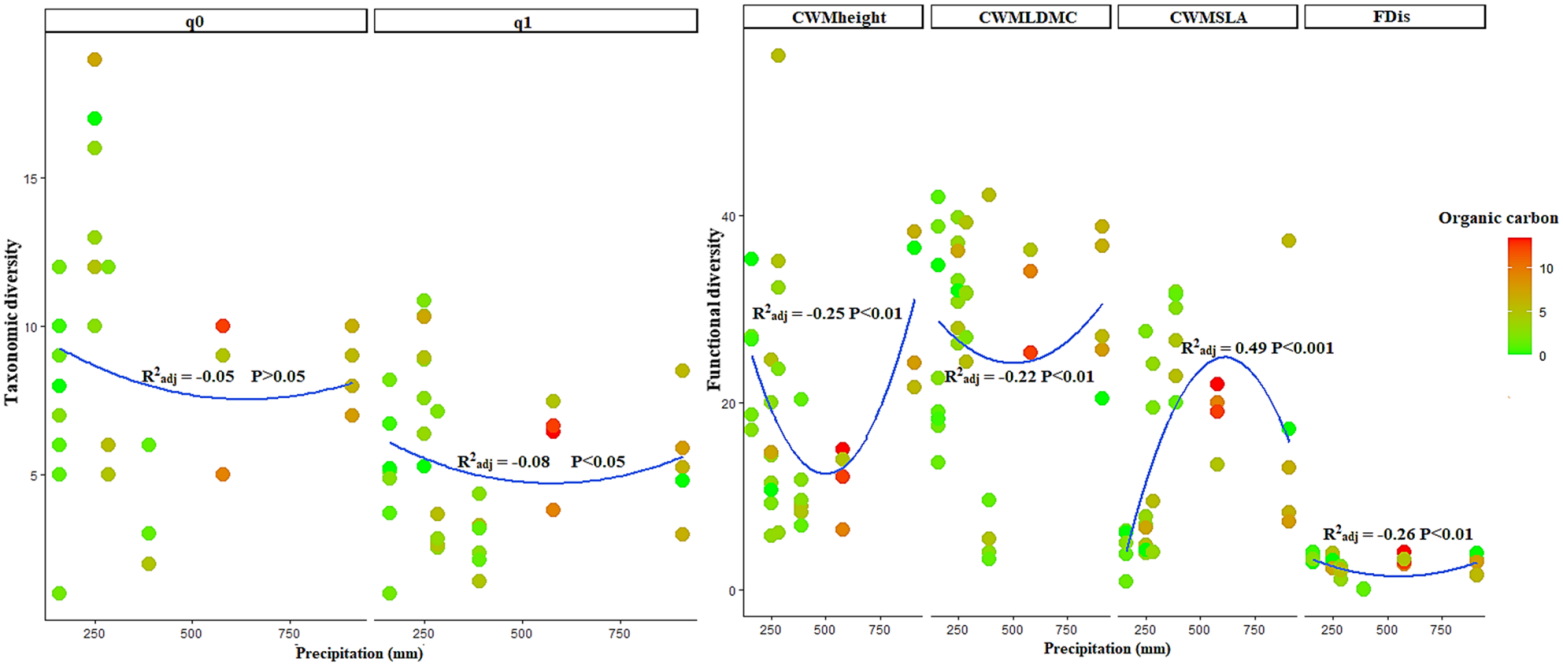
Variation in taxonomic (q_0_ and q_1_ in left panels) and functional (CWMheight, CWMLDMC, CWMSLA and FDis in right panels) diversity in rocky outcrops across precipitation and organic carbon factors.

The taxonomic diversity similarly changed across precipitation and soil factors in both outcrops and their surrounding rangelands (Figs. 1, 2, Appendix S2). In this regard, we found a decrease in taxonomic diversity especially under intermediate levels of precipitation and soil factors (organic carbon in outcrops and phosphorus in rangelands) in both the environments (please see q0 and q1 panels in Figs. 1 and 2 and Appendix S2). Variations in taxonomic indices across environmental factors were not significant in outcrops (except for q1 index), whereas taxonomic indices significantly responded to precipitation and phosphorus in surrounding rangelands. In general, rangeland ecosystems had more species richness and diversity than outcrops (Fig. 1; q0 and q1 panels).

Functional indices showed significant nonlinear variations across precipitation and soil factors in both outcrops and their surrounding rangelands (Figs. 1, 2). In rangelands, functional diversity indices including CWM-plant height (CWM_Height_), CWM_SLA_ and FDis significantly decreased under moderate levels of precipitation and phosphorus gradients (Fig. 1; CWM_Height_, CWM_SLA_, FDis panels). In contrast, an increase for CWM_LDMC_ was observed under intermediate levels of precipitation and phosphorus gradients (Fig. 1; CWM_LDMC_ panel). In outcrops, CWM_Height_, CWM_LDM_c and FDis indices significantly decreased under moderate levels of precipitation and organic carbonWhereas, we found a strong and significant increase for CWM_SLA_ across organic carbon and precipitation gradients. Overall, our results indicated more consistent and significant variations for functional diversity indices than taxonomic indices in both outcrops and their surrounding rangelands. (Figs. 1, 2; R^2^ and P-value for FDis, CWM_LDMC_, CWM_height_ and CWM_SLA_ in outcrops and rangelands). In addition, in outcrops, variations in functional diversity indices across environmental factors were stronger than rangelands, with more negative and significant effects of precipitation and organic carbon on FDis, CWM_LDMC_ and CWM_height_ Whereas, CWM_SLA_ under moderate levels of precipitation and organic carbon (Fig. 2). In rangeland sites, although all functional diversity indices studied exhibited negative trends across precipitation and phosphorus factors (except in FDis index), we found more negative and significant variations of CWM_SLA_ across environmental gradients (Fig. 1).

### Relative importance of precipitation and soil factors on taxonomic and functional diversity

**Figure 3:**
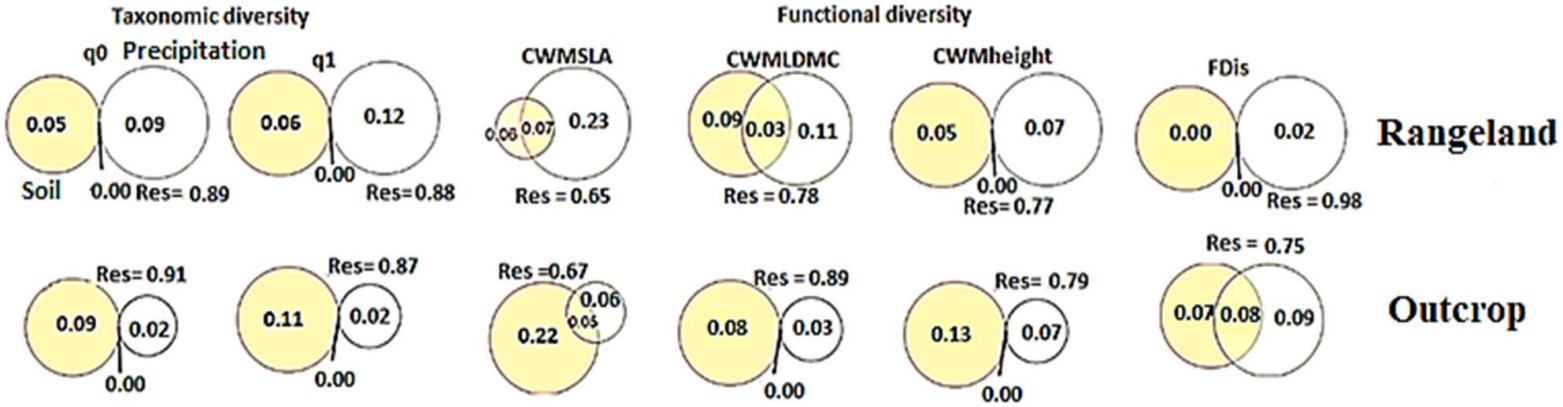
The relative contribution of environment (in yellow; i.e. soil factors; organic carbon (OC) for rocky outcrops, Phosphorus (P) for surrounding rangelands) and precipitation (in white) to taxonomic (q0 and q1 indices) and functional (CWMALA, CWMLDMC, CWMheight and FDis) in surrounding rangelands (up) and rocky outcrops (down). Values represent the adjusted R^2^‐values.

Our results indicated relative importance of precipitation and soil factors on structuring taxonomic and functional diversity in outcrops and their surrounding rangelands (Fig. 3). In outcrops, soil factors including organic carbon) Fig. 3; yellow circles) explained higher contributions of variation in taxonomic (q0 (9%), q1 (11%)) and functional (CWM_SLA_ (22%), CWM_LDMC_ (8%), CWM_Height_ (13%) and FDis (7%)) diversity than precipitation (Fig. 3; Outcrop panel, yellow circles). Indeed, Precipitation was not an appropriate factor in explaining the changes for biodiversity indices in outcrops. In contrast, precipitation explained a large contribution of plant biodiversity in surrounding rangelands. In this regard, precipitation (Fig. 3; white circles) explained higher proportion of variations in taxonomic (q0 (9%), q1 (12%)) and functional (CWM_SLA_ (23%), CWM_LDMC_ (11%), CWM_Height_ (7%) and FDis (2%)) diversity than soil factors such as phosphorus (yellow circles) (Fig. 3; Rangeland panel). Therefore, precipitation and soil factors including organic carbon had more consistent and significant effects on biodiversity indices in rangelands and outcrops, respectively (Fig. 3; Rangeland and Outcrop panels).

## Discussion

We found environmental factors such as precipitation and soil factors significantly influenced taxonomic and functional diversity in outcrops and their surrounding rangelands. However, there are differences in the trend and extend of such variations along the gradients.

### Plant diversity indices along the precipitation and soil factors gradients

Throughout the system under study, hill numbers as taxonomic diversity indices showed in the rangeland a greater diversity of outcrops. Taxonomic variability showed similar changes and decreased along soil and precipitation gradient in outcrop and rangeland. In the outcrops and their surrounding rangelands, taxonomic diversity was decreased under moderate levels of variation across environmental factors. Taxonomic diversity generally can be affected by soil fertility, topographic factors, and even biotic factors^74^. However, variations in some soil factors can significantly influence presence of plant species based on their realized niches especially in more severe spatial scales^44,75^. Therefore, in outcrops with more severe environmental conditions than rangelands, we found a more significant effects of soil factors than climatic factors such as precipitation on structuring taxonomic diversity (see Fig. 3; Outcrop panel). While, in rangelands, important effects of climatic factors such as precipitation could determine shaping taxonomic diversity and presence of plant species in plant communities (see Fig. 3; Rangeland panel). On the other hands, more significant effects of phosphorus factors than other soil factors studied can be related to competition between plant species in absorption of phosphorus of deeper layers of soil^76^. However, absorption of phosphorus is correlated to the amount of precipitation, as a decrease in precipitation can lead to an increase in phosphorus (due to the death of some plant tissues under an increase in aridity index)^77^. In a study across the precipitation and soil fertility in northeastern Iran^78^, a decrease in taxonomic diversity was also observed by increasing mean annual precipitation. Huston^79^ believes variations in taxonomic diversity across precipitation gradient are associated to mechanisms regulating the species coexistence and variation in functional diversity^80,81^.

We found nonlinear changes in functional diversity in response to environmental factors, under harsh outcrop conditions and in their surrounding rangeland sites. More negative and significant trends of variation in functional diversity (except for CWM_SLA_) across environmental factors was observed in outcrops than rangeland sites. Such reduction in functional diversity can be due to presence of low-yielding species that are functionally similar^12,82^. Climatic factors coupled by variations in soil factors reduce biodiversity by providing different adaptations to deal with water and nutrient stress. Therefore, such adaptations may maintain the coexistence of species with different levels of functional traits^80,81^. In addition, spatial (resource niche) and temporal heterogeneity (temporal climate diversity) in arid conditions provide heterogeneous niches for presence of species with different levels of functional traits^83^. Changes in functional diversity have different trends in rangeland and outcrop with increasing precipitation. While is observed, an increase in the presence of species with different functions especially in the middle of soil and precipitation gradient in rangeland. This shows that the functional differentiation among species and species diversity can very rather independently to each other. It has been noted that environmental filters might limit species diversity and composition to a given range of functional characteristics^35^. The reason for the presence of species with high functional convergence is due to the micro-habitat role of the outcrops in facilitating functional groups with similar characteristics in the outcrops^77^. Functional facilitation of species can also occur indirectly under the influence of environmental factors. Functional divergence of species can be due to the prominent role of competition or equilibrium interaction between competition and facilitation, especially in the middle of soil and precipitation gradient in rangeland. Competition between similar functional groups due to having similar niches causes plant species with different functional domains to be present in a community^12^.

### Changes in ecological and functional traits of plant community

Changes in community traits showed a similar trend across precipitation and soil factors gradients in outcrops and rangelands. However, analysis of the measured plant traits showed different functional syndromes of traits observed at the community level along the precipitation and soil factors gradients. The average specific leaf area and plants height were low in the drier sites but increased along the precipitation and Phosphorus gradients in rangelands. However, in outcrops community height and leaf dry matter content decreased and specific leaf area increased in the middle gradient to increasing precipitation and organic carbon.

In the arid ecosystems, harsh environmental conditions such as low precipitation and high temperatures induces carbon storage, which result in lower leaf area and specific leaf area as compared with plant growing under the more favorable environmental conditions^84^. Previous studies have also shown that SLA and LA are often positively correlated with photosynthesis and growth rate and evapotranspiration rate^85^, and plants with high SLA and LA strategies and high evapotranspiration rates are often unable to tolerate drought stress^86,87^. Our results also indicated plant height increase along the precipitation gradient from arid-steppe towards dense temperate forest. Plant height is positively related to the competitive ability in obtaining light in plant communities^88^. On the other hand, high leaf dry matter content that we found in drier areas indicate species adaptation and their increased resistance to environmental stresses^89–91^. like previous researches in our study also, soil factors (i.e. phosphorus) was more associated with plant traits^92^, leaf area, specific leaf area, plant height, and leaf dry matter content positively correlated with available soil phosphorus and nitrogen^93^, soil factors influenced plant traits and species diversity and richness^94,95^.

### Role of environmental factors on plant diversity indices

The results of biodiversity analysis on variation partitioning showed that both precipitation and soil properties influenced taxonomic and functional diversity. However, the contribution of soil factors in outcrops was more than rainfall. Whereas, in the rangeland sites, precipitation was the most important factor in structuring of taxonomic and functional diversity.

Richness indices, taxonomic and functional diversity were affected by organic carbon in the outcrop. This shows that soil fertility can play an essential role in increasing these indices. Soil fertility has a significant effect on controlling the amount of moisture and nutrients available to plants, which have profound effects on vegetation composition changes^96^. The factors that directly affect taxonomic diversity, later on may affect other abiotic factors^97^. Similarly in granitic and gneissic outcrops of south-eastern Brazil, taxonomic and functional diversity were significantly affected by abiotic factors of soil factors and soil depth^98^.

In the rangeland (i.e. soil covered landscapes), Precipitation factor was the most important factor affecting richness, Hill diversity and functional dispersion. In general, Hill plant diversity was more affected by soil properties (organic carbon) in outcrop, while the average annual precipitation was more important in rangeland^11,13,99^. Nevertheless for functional dispersion, the contribution of precipitation factor (soil vs. precipitation) was more than phosphorus and organic carbon in rangeland and outcrops^100^.

Plant traits were more associated with organic carbon than climatic factors in outcrop. Indices of the community single traits (i.e. specific leaf area, plant height, and leaf dry matter content) were more explained by organic carbon than precipitation in outcrop^101^. Rocky outcrops showed more correlation to soil factors, and climatic factors such as precipitation had a negligible effect on explaining biodiversity and ecological traits. In rangeland plant traits were more affected with precipitation factors than soil phosphorus. Main factors affecting plant Leaf Dry Matter Content (LDMC) in the outcrop was organic carbon, but in the nearby covered lands they were mostly affects by phosphorus. The high observed changes in LDMC are likely related to an efficient nutrient conservation strategy^102,103^. In addition, leaves with a high dry matter content may maintain torque stress with relatively more minor water potential and increase drought resistance and freezing resistance^104,105^. In general, plant height and their growth period were decreased by increasing soil Na and K that salinity may be due to disturbances in nutrient uptake, disturbance of ionic balance or reduction of soil water potential and osmotic stress, or changes in enzymes affecting the plant photosynthetic activity apparatus^106^.

## Conclusions

We investigated changes in plant taxonomic and functional diversity, along the precipitation gradients and soil properties in rocky outcrops and adjacent covered landscapes. According to our results, changes in soil properties (organic carbon) more affected taxonomic, functional diversity and functional traits in outcrop. Whereas, in the rangeland sites, precipitation was the most important factor in structuring of taxonomic and functional diversity. Whereas, similar environmental factors (Phosphorus and organic carbon and precipitation) regulated taxonomic diversity of rocky outcrops and nearby rangelands, functional diversity showed greater diversity of drought adapted traits at the community level of rocky outcrops. Therefore, our results highlight important role of micro-scale environmental factors such as presence of critical species (keystone species) and/or effects of microhabitats on plant community composition and diversity facet along the environmental gradients. For future researches, considering other climatic variables (seasonal precipitation and minimum and maximum annual temperatures, etc.) and topographic factors (height, aspect, and percentage of slope), and the effects of biotic interactions and their relative importance along abiotic factors, will bring more insights on plant diversity of the mountainous (here out-crop versus nearby soil covered landscapes) dryland ecosystems.

## Methods

### Study region

**Figure 4:**
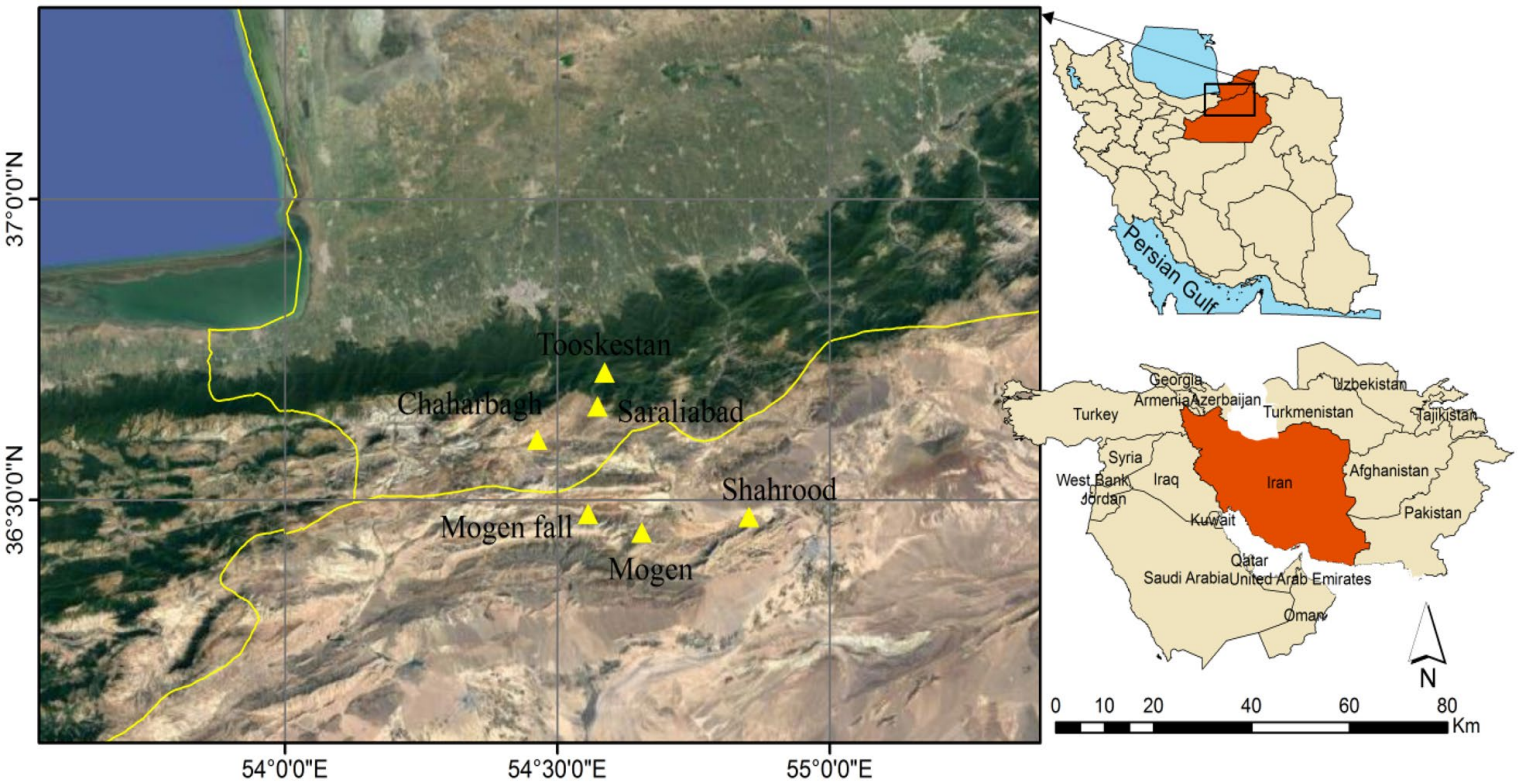
Map of the study area in Northeast of Iran, in which site locations are shown. The following map has been generated in َArcmap ver. 10.2 (https://www.esri.arcGIS.desktop.com).

The study area is located in southwest Asia and north of Iran, crosses Alborz mountains from arid steppe rangeland in Shahroud to temperate forests in Gorgan. The study areas were selected on calcareous geological formation and north facing slopes. Six sites were selected (Fig. 4), with the annual precipitation (mm) from 160 (Shahroud), 250 (Mojen), 285 (Mojen waterfall), 390 (Chaharbagh), 580 (Sar Ali Abad), to 910 (Tooskestan) (Appendix S1). The selected homogeneous study sites. They were located in in terms north-facing slopes, calcareous geological formation, main land use as rangelands However, they were different depending in terms of dominant plant species (shrubs, perennial forbs and shrub, and large trees), average yearly precipitation [160 mm (Shahroud) − 910 mm (Tooskestan)], elevation [800 m (Shahroud) − 2700 m (Sar Ali Abad)] and soil factors including pH [7.9 (Tooskestan) − 8.7 (Shahroud)m], EC [74 (Mojen) − 338 (Tooskestan) (dS m^−1^)], K [216 (SarAliAbad) – 860 (Chaharbagh) (mg kg^−1^)], N [0.06 (Shahroud) − 0.69 (SarAliAbad) (mg kg^−1^)], Lime (%) [10.5 (Chaharbagh) − 26.27 (Shahroud)], Na [24 (Tooskestan) − 86 (Shahroud) (mg kg^−1^)], P [4 (Mojen) − 25 (Chaharbagh) (mg kg^−1^)], OC [0.4 (Shahroud) − 16 (Tooskestan) (%)], Clay [22 (Shahroud) − 60 (Chaharbagh) (%)], Silt [28(Shahroud) − 60 (Chaharbagh) (%)] and Sand [4 (Tooskestan) − 44 (Shahroud) (%)] (Appendix S1). We confirm that our study and sampling methods conducted comply with local and national regulations or guidelines.

### Data collection

Vegetation sampling was done in a systematic randomized method in which six sites (i.e. Shahroud, Mojen, Mojen waterfall, Chaharbagh, Sar Aliabad, and Tooskestan) were systematically established along the precipitation and soil gradients and in two landscapes (i.e. rocky outcrops and their surrounding rangelands). Then, within each landscape in each site, 15 1 m^2^ quadrats were randomly established (15 × 6 = 90 quadrats in total for each landscape). Sampling was conducted in May–June 2017, but complementary sampling was accomplished as same months in 2018. The distance between sampling units were approximately 50–100 m. In each quadrat, the species abundance, canopy cover, climatic, soil and topographic information were recorded. Plant specimens were collected to identify and measure functional trait (10 individuals of each species) in each plot. They were immediately, packed in paper bags, sealed in plastic bags, and transferred to the laboratory.

### Plant functional information

Functional diversity is assessed based on functional traits. Functional traits describe indicators of competition, growth, adaptation, establishment, and environmental variables. As a result, 10 qualitative and quantitative traits were selected based on the objectives of study. They were: Plant height, Seed mass, Leaf area (LA), Specific leaf area (SLA), Leaf dry-matter content (LDMC), Life form, Life span, Clonality, Spinences and Palatability^107^. To measure these traits, 10 individuals of each species were collected and placed in plastic bags to retain moisture and transferred to the laboratory. Plant height is the shortest distance between the upper foliage boundary and ground level^108^.

We determined the leaf area using a digital scanner and Leaf Area Measurement v1.3 software (Andrew Askew, University of Shefeld, UK). Leaf fresh matter content was obtained from saturated leaves, and leaf dry matter content was determined after drying for 72 h in an oven at 70 °C. For qualitative traits, plant life forms were coded into five classes: phanerophytes (Ph), chamaephytes (Ch), hemicryptophytes (He), geophyte (Ge), and therophytes (Th), using Raunkiaer’s^109^ classification. Clonality was expressed as the presence/absence of clonal reproduction of individual species via assessing of rhizomes or runners. Life span was also divided into annual and perennial. Thorns were also classified based on the presence or absence of thorns.

### Soil collection and processing

Soil samples (~ 500 g) were taken at a 0–20 cm depth, placed in a polyethylene bag, labeled, and transported to the laboratory. The following properties were measured in each plot in the outcrop (39 samples) and their surrounding rangelands (30 samples): pH, electrical conductivity (EC), organic carbon (OC), Sodium (Na), total nitrogen (N), Potassium (K), phosphorus (P) and soil texture components including lime, silt, sand, and clay percentage. Bykas hydrometric method^110^ was used to determine soil texture. Total nitrogen (N) was determined by the Kjeldahl method^111^. Organic carbon (OC) was analyzed by the Walkley and Black^112^ method^113^. Soil electrical conductivity (EC) and acidity (pH) were determined using pH and EC meters. Total potassium (K) and sodium (Na) were analyzed by flame atomic absorption spectrophotometer^114^. Absorbable phosphorus was analyzed by the Olsen method. The percentage of total lime was measured by titration method with 0.01 N NaOH^115^. Finally, we prepared a matrix of 12 independent variables (i.e. annual precipitation, sodium (Na), potassium (K), pH, electrical conductivity (EC), lime, total nitrogen (N), phosphorus (P), organic carbon (OC), clay, sand, and silt ) and used in further analyses.

### Statistical analysis

#### Measures of taxonomic and functional diversity

We measured the taxonomic diversity using RaoQ index. In this regard, the first three Hill numbers of RaoQ index were selected to estimate species richness (q = 0), the exponential of Shannon's entropy (q = 1; referring to Shannon diversity) and the inverse of Simpson's concentration (q = 2; referring to Simpson diversity). This analyze was computed using R package hillR^116^.

Functional diversity was calculated using the community weighted means index (CWM) and multi-trait functional diversity indices such as FDis. CWM traits were calculated as mean trait values for each vegetation plot, weighted by the relative abundances of species with that particular trait values^117,118^. The community‐weighted means (CWM) for each trait and community sample were calculated as ΣPi × Trait i, where Pi is the relative abundance of species i in the community sample and j trait i is the trait value. Further, mean values of individual traits (height, seed mass, leaf area, clonality, annual–perennial life history) were calculated for each vegetation plot. Eventually, the mean trait values per plot (weighted by the relative abundances of species) were essential in the analyses. Functional dispersion (FDis) was calculated based on Laliberte and Legendre^119^ procedure. We chose FDis among the many functional diversity metrics because it describes the distribution of species in trait space, can be used for multiple traits, is not strongly influenced by outliers, and is independent of species richness. We calculated FDis using the “FD” function in R package FD^119^.

#### Statistical analyses

We analyzed variation in taxonomic and functional diversity relative to precipitation, soil factors and their interactions in both outcrops and their surrounding rangelands. Some of the environmental factors were highly correlated with each other and could induce multicollinearity in our models. To avoid this, environmental variables with |*r*|> 0.7 were considered highly correlated^120^ hence they were removed from analysis to to avoid model predictions induced by multicollinearity among environmental variables (Appendix S1 and S4). Further, we analyzed multicollinearity amongst the remaining variables using variance inflation factors (VIF) (function *vif()* in the package ‘car’^121^ and variables with VIF scores > 10 were considered to be highly collinear^122^ and removed from our environmental matrix. For outcrops, only seven variables (annual precipitation, OC, Clay, Silt, P, elevation and limestone were selected and used on subsequent analyses. Annual precipitation, pH, P, elevation, Silt, Sand and K were selected as non-correlated variables for surrounding rangelands. To further simplify our models, we used a forward selection procedure (function forward.sel () in the package “packfor”^123^), keeping only those environmental variables selected in the most parsimonious models for taxonomic and functional diversity with respect to the usual alpha significance level and the adjusted coefficient of multiple determination (R^2^adj) calculated using all explanatory variables^124^. Annual precipitation and Organic Carbon (OC) were selected as most important factors influencing the plant biodiversity indices in outcrops, whereas Phosphorus (P) and annual precipitation were the ones selected in surrounding rangelands.

With the final list of predictors, we developed linear regression models with precipitation and Phosphorus in outcrops and Annual precipitation and Limestone in surrounding rangelands as explanatory variables and q = 0, q = 1, FDis, CWM SLA, CWM LA, CWM LDMC and CWM plant height as response variables. In addition to the linear trend, we also tested for non-linear trends of biodiversity facets with our explanatory predictors by developing non-linear regression models. Then, we compared these models using second-order Akaike information criteria (AIC) and R^2^_adj_ values in both the ecosystems (see details in Appendix S3 in Supporting Information). Finally, the best models were plotted and their R^2^_adj_ values were obtained using the package ‘vegan”^125^.

To assess the impacts of precipitation, soil factors, and their interactions on biodiversity indices at rangeland and outcrop sites, we performed variation partitioning based on partial linear regression using the “varpart” function^125^. The total percentage of variation explained was divided into unique and shared contributions for two predictors: (1) precipitation (white fraction), (2) soil (i.e. phosphorus in rangeland and organic carbon in outcrop) (yellow fraction), and (3) shared contributions of both factors (shared area between yellow and white fractions). Res. Value indicated residuals (i.e. the part of plant biodiversity which was not explained by the studied explanatory variables). Analyses were conducted in R ver. 4.0.0, and figures were produced using the ggplot2 package^126^.

## Supplementary Information

Supplementary Information.

## Data Availability

All data generated or analyzed during this study are included in this published article [Appendix S5 and S6 in supporting information].
