# Author Correction: Changes in plant biodiversity facets of rocky outcrops and their surrounding rangelands across precipitation and soil gradients

**DOI:** 10.1038/s41598-022-16723-0

**Published:** 2022-07-22

**Authors:** Fahime Rafiee, Hamid Ejtehadi, Mohammad Farzam, Habib Zare, Maral Bashirzadeh

**Affiliations:** 1 Quantitative Plant Ecology and Biodiversity Research Lab, Department of Biology, Faculty of Science, Ferdowsi University of Mashhad, Mashhad, Iran; 2 Department of Range and Watershed Management, Faculty of Natural Resources and Environment, Ferdowsi University of Mashhad, Mashhad, Iran; 3 Botanical Garden of Nowshahr, Research Institute of Forests and Rangelands, Agricultural Research, Education and Extension Organization, AREEO, Tehran, Iran

Correction to: *Scientific Reports* 10.1038/s41598-022-13123-2, published online 30 May 2022

The original version of this Article contained an error in Affiliation 3, which was incorrectly given as ‘Herbarium of Nowshahr Botanical Garden, Research Center of Agriculture and Natural Resources of Mazandaran, Mazandaran, Iran’.

The correct affiliation is listed below.

Botanical Garden of Nowshahr, Research Institute of Forests and Rangelands, Agricultural Research, Education and Extension Organization, AREEO, Tehran, Iran

The original Article has been corrected.

