# Supplementary material for: Changes in plant biodiversity facets of rocky outcrops and their surrounding rangelands across precipitation and soil gradients
Source: Sci Rep. 2022 May 30;12:9022. doi: 10.1038/s41598-022-13123-2 (PMC9151709; doi:10.1038/s41598-022-13123-2)
Supplement: Supplementary file 1 — Supplementary Information. [file 41598_2022_13123_MOESM1_ESM.docx]

**Supplementary information:**

Three supplementary files are associated with this paper:

**Appendix S1**. Essential characteristics of the study area and environmental factors measured along the rainfall and soil fertility gradients.

| Soil factors | Mean annual precipitation(mm) | Elevation (m) | Coordinates | Location |
| --- | --- | --- | --- | --- |
| pH (8.3-8.7) m, EC (69.3-115.7) (dS m -1), Lime (16.1-26.3)%, Clay(20-34)%,Silt(34-60)%, Sand(12-44)%, Na(33.9-86.6) (mg kg-1), K ( 490.4-675.9) (mg kg-1), N(0.06-0.15) (mg kg-1), P(5-17) (mg kg-1), OC(0.43-2.4)% | 160 | 1688 | 54° 51' 6.7"E- 36° 28' 6.9"N | Shahroud |
| pH ( 8.2-8.6)m, EC(74.5-154.6) (dS m -1), Lime( 14.5-26.2)% ,Clay(30-48)%, Silt(28-56)%, Sand( 4-34)%, Na(26.1-83.5) (mg kg-1), K( 484.4-842.7) (mg kg-1), N(0.09-0.27) (mg kg-1), P(4-15) (mg kg-1), OC( 1.95-7) % | 250 | 2236 | 54° 39' 13.5"E-36° 26' 8.2"N | Mojen |
| pH (8.2-8.5), EC( 87-138) (dS m -1),Lime( 16-26)%, Clay( 26-56)%, Silt( 28-56)%, Sand( 10-44)%, Na(24-72) (mg kg-1), K( 275-765) (mg kg-1), N( 0.08-0.2) (mg kg-1), P( 4-13) (mg kg-1), OC(1.09-5.3)%. | 285 | 2338 | 54° 33' 14.1"E-36° 28' 43.5"N | Mojen fall |
| pH ( 8.3-8.6)m, EC(91-148) (dS m -1), Lime( 10-26)%, Clay( 30-60)%, Silt( 32-56)%,Sand( 12-34)%, Na(39-53) (mg kg-1), K( 386-860) (mg kg-1), N( 0.08-029) (mg kg-1),P( 5-25) (mg kg-1), OC( 1.2-5.4)%. | 390 | 2078 | 54° 27' 48.1"E-36° 36' 1.58"N | Chaharbagh |
| pH ( 8.1-8.6)m,EC( 76-142) (dS m -1),Lime( 11.3-26.2)%, Clay( 24-46)%, Silt( 36-50)%, Sand( 10-40)%,Na( 36-91) (mg kg-1),K( 216-666) (mg kg-1),N( 0.02-0.69) (mg kg-1), P( 4-22) (mg kg-1),OC( 1.9-13.4)%. | 580 | 2655 | 54° 34' 25.2"E-36° 39' 19.69"N | Sar aliabad |
| pH ( 7.9-8.8)m,EC( 90-338) (dS m -1), Lime( 14-26)%, Clay( 26-44)%,Silt( 32-44)%, Sand( 4-34)%,Na( 24-79) (mg kg-1),K( 529-700) (mg kg-1),N(0.2-0.7) (mg kg-1),P(4-13) (mg kg-1),OC( 5-16)%. | 910 | 882 | 54° 34' 25.2"E-36° 42' 49.8"N | Tooskestan |

**Appendix S2.** Results of linear and nonlinear regression models on the effect of precipitation and soil factors on taxonomic (q0,q1 Hill) and functional ( Functional dispersion (FDis), Community-Weighted mean Specific Leaf Area ( CWM_SLA)_ , Community-Weighted mean Leaf Dry Matter Content ( CWM_LDMC_ ) and Community-Weighted mean Height( CWM_Height_ ) indices ) diversity along precipitation and soil factors gradient in rocky outcrop and rangeland.

| **lm** | **landscape** | **response** | **explanatory** | **R^2^** | **P** | **AIC** |
| --- | --- | --- | --- | --- | --- | --- |
| linear | Outcrop | q0 | Precipitation + OC | 0.02597 | 0.6394 | 218.4821 |
| nonlinear | Outcrop | q0 | Precipitation + OC | 0.05822 | 0.7397 | 221.236 |
| linear | Outcrop | q1 | Precipitation + OC | 0.02732 | 0.642 | 173.8952 |
| nonlinear | Outcrop | q1 | Precipitation + OC | 0.09485 | 0.5434 | 175.3768 |
| linear | Rangeland | q0 | Precipitation + P | 0.06567 | 0.4136 | 183.0986 |
| nonlinear | Rangeland | q0 | Precipitation + P | 0.07682 | 0.7365 | 186.7502 |
| linear | Rangeland | q1 | Precipitation + P | 0.0273 | 0.2576 | 558.6042 |
| nonlinear | Rangeland | q1 | Precipitation + P | 0.05243 | 0.2651 | 559.9607 |
| linear | Outcrop | FDis | Precipitation + OC | 0.01574 | 0.7697 | 125.0558 |
| nonlinear | Outcrop | FDis | Precipitation + OC | 0.4955 | 0.000215 | 104.9929 |
| linear | Outcrop | CWMSLA | Precipitation + OC | 0.1495 | 0.06916 | 269.6848 |
| nonlinear | Outcrop | CWMSLA | Precipitation + OC | 0.4929 | 0.000232 | 255.0666 |
| linear | Outcrop | CWMLDMC | Precipitation + OC | 0.08558 | 0.2285 | 281.4932 |
| nonlinear | Outcrop | CWMLDMC | Precipitation + OC | 0.2259 | 0.08501 | 279.4944 |
| linear | Outcrop | CWMHeight | Precipitation + OC | 0.06021 | 0.359 | 284.9048 |
| nonlinear | Outcrop | CWMHeight | Precipitation + OC | 0.2657 | 0.04266 | 280.0208 |
| linear | Rangeland | FDis | Precipitation + P | 0.01882 | 0.3419 | 928.0693 |
| nonlinear | Rangeland | FDis | Precipitation + P | 0.02905 | 0.3454 | 928.8535 |
| linear | Rangeland | CWMSLA | Precipitation + P | 0.3863 | 0.00175 | 203.7055 |
| nonlinear | Rangeland | CWMSLA | Precipitation + P | 0.4392 | 0.006062 | 205.0907 |
| linear | Rangeland | CWMLDMC | Precipitation + P | 0.05365 | 0.4883 | 225.0111 |
| nonlinear | Rangeland | CWMLDMC | Precipitation + P | 0.1194 | 0.5291 | 226.9232 |
| linear | Rangeland | CWMHeight | Precipitation + P | 0.1073 | 0.2285 | 221.0331 |
| nonlinear | Rangeland | CWMHeight | Precipitation + P | 0.132 | 0.3069 | 222.2193 |

**Appendix S3**. The most critical environmental factors affected taxonomic and functional diversity on based R^2^adj in forward selection.

| Landscape | Function | explanatory | response | R2 | P-value |
| --- | --- | --- | --- | --- | --- |
| outcrop | forward sel | oc | Biodiversity | 0.291729 | 0.002 |
| outcrop | forward sel | precipitation | Biodiversity | 0.383633 | 0.009 |
| rangeland | forward sel | precipitation | Biodiversity | 0.340102 | 0.001 |
| rangeland | forward sel | P | Biodiversity | 0.435471 | 0.003 |

**Appendix S4.**

Correlation between soil factors considered using Pearson correlation analysis.

|  | Ph | EC | caco3 | clay | silt | sand | Na | K | N | P | oc |
| --- | --- | --- | --- | --- | --- | --- | --- | --- | --- | --- | --- |
| Ph | 1 | -0.11453 | 0.134233 | -0.1285 | 0.124856 | -0.15682 | -0.07365 | -0.07994 | -0.45232 | 0.173464 | -0.4561 |
| EC | -0.11453 | 1 | 0.157679 | -0.21502 | -0.27068 | 0.121244 | -0.15495 | -0.14375 | 0.559842 | -0.18066 | 0.616417 |
| caco3 | 0.134233 | 0.15768 | 1 | -0.20141 | -0.0785 | 0.287456 | 0.073524 | 0.087793 | -0.04676 | 0.092415 | -0.05731 |
| clay | -0.1285 | -0.21502 | -0.20141 | 1 | 0.533847 | -0.14573 | 0.101488 | 0.150018 | -0.15342 | -0.01262 | -0.11579 |
| silt | 0.124856 | -0.27068 | -0.0785 | 0.533847 | 1 | -0.06603 | -0.03345 | 0.07417 | -0.11386 | 0.041657 | -0.10759 |
| sand | -0.15682 | 0.121244 | 0.287456 | -0.14573 | -0.06603 | 1 | 0.120819 | 0.195013 | 0.225508 | -0.01974 | 0.17186 |
| Na | -0.07365 | -0.15495 | 0.073524 | 0.101488 | -0.03345 | 0.120819 | 1 | 0.724989 | -0.12069 | 0.157428 | -0.15926 |
| K | -0.07994 | -0.14375 | 0.087793 | 0.150018 | 0.07417 | 0.195013 | 0.724989 | 1 | -0.10245 | 0.046239 | -0.13622 |
| N | -0.45232 | 0.559842 | -0.04676 | -0.15342 | -0.11386 | 0.225508 | -0.12069 | -0.10245 | 1 | -0.27564 | 0.963781 |
| P | 0.173464 | -0.18066 | 0.092415 | -0.01262 | 0.041657 | -0.01974 | 0.157428 | 0.046239 | -0.27564 | 1 | -0.30512 |
| oc | -0.4561 | 0.616417 | -0.05731 | -0.11579 | -0.10759 | 0.17186 | -0.15926 | -0.13622 | 0.963781 | -0.30512 | 1 |

**Appendix S5**. Raw data used to picture variations in taxonomic (q0 and q1) and functional (FDis, CWMSLA, CWMLDMC, CWMheight) diversity across precipitation and organic carbon (OC) in rocky outcrops.

| number | OC | Precipitation | plant biodiversity | class |
| --- | --- | --- | --- | --- |
| 1 | 1.131 | 160 | 5 | q0 |
| 2 | 1.52 | 160 | 9 | q0 |
| 3 | 0.97 | 160 | 6 | q0 |
| 4 | 1.75 | 160 | 1 | q0 |
| 5 | 0.438 | 160 | 10 | q0 |
| 6 | 1.79 | 160 | 12 | q0 |
| 7 | 0 | 160 | 8 | q0 |
| 8 | 2.45 | 160 | 7 | q0 |
| 9 | 2.65 | 250 | 10 | q0 |
| 10 | 3.19 | 250 | 13 | q0 |
| 11 | 3 | 250 | 16 | q0 |
| 12 | 2.96 | 250 | 12 | q0 |
| 13 | 2.106 | 250 | 17 | q0 |
| 14 | 4.99 | 250 | 12 | q0 |
| 15 | 0 | 250 | 17 | q0 |
| 16 | 7.02 | 250 | 19 | q0 |
| 17 | 2.14 | 285 | 12 | q0 |
| 18 | 3.15 | 285 | 5 | q0 |
| 19 | 4.87 | 285 | 5 | q0 |
| 20 | 3.081 | 285 | 6 | q0 |
| 21 | 5.3 | 285 | 6 | q0 |
| 22 | 5.26 | 390 | 6 | q0 |
| 23 | 2.49 | 390 | 6 | q0 |
| 24 | 2.61 | 390 | 3 | q0 |
| 25 | 4.75 | 390 | 2 | q0 |
| 26 | 1.28 | 390 | 3 | q0 |
| 27 | 1.209 | 390 | 6 | q0 |
| 28 | 13.4 | 580 | 10 | q0 |
| 29 | 9.36 | 580 | 5 | q0 |
| 30 | 12.4 | 580 | 10 | q0 |
| 31 | 4.87 | 580 | 9 | q0 |
| 32 | 6.43 | 910 | 10 | q0 |
| 33 | 6.24 | 910 | 8 | q0 |
| 34 | 0 | 910 | 7 | q0 |
| 35 | 7.68 | 910 | 7 | q0 |
| 36 | 5.65 | 910 | 9 | q0 |
| 37 | 1.131 | 160 | 5 | q0 |
| 38 | 1.52 | 160 | 5.107462 | q1 |
| 39 | 0.97 | 160 | 3.69149 | q1 |
| 40 | 1.75 | 160 | 1 | q1 |
| 41 | 0.438 | 160 | 6.705189 | q1 |
| 42 | 1.79 | 160 | 8.184661 | q1 |
| 43 | 0 | 160 | 5.218197 | q1 |
| 44 | 2.45 | 160 | 4.850848 | q1 |
| 45 | 2.65 | 250 | 7.562765 | q1 |
| 46 | 3.19 | 250 | 6.389848 | q1 |
| 47 | 3 | 250 | 8.875432 | q1 |
| 48 | 2.96 | 250 | 10.35417 | q1 |
| 49 | 2.106 | 250 | 10.84946 | q1 |
| 50 | 4.99 | 250 | 8.95776 | q1 |
| 51 | 0 | 250 | 5.267109 | q1 |
| 52 | 7.02 | 250 | 10.29991 | q1 |
| 53 | 2.14 | 285 | 7.124047 | q1 |
| 54 | 3.15 | 285 | 2.522911 | q1 |
| 55 | 4.87 | 285 | 2.641892 | q1 |
| 56 | 3.081 | 285 | 2.845204 | q1 |
| 57 | 5.3 | 285 | 3.660564 | q1 |
| 58 | 5.26 | 390 | 3.303058 | q1 |
| 59 | 2.49 | 390 | 4.348513 | q1 |
| 60 | 2.61 | 390 | 2.363525 | q1 |
| 61 | 4.75 | 390 | 1.417714 | q1 |
| 62 | 1.28 | 390 | 2.12382 | q1 |
| 63 | 1.209 | 390 | 3.173848 | q1 |
| 64 | 13.4 | 580 | 6.433076 | q1 |
| 65 | 9.36 | 580 | 3.793634 | q1 |
| 66 | 12.4 | 580 | 6.656927 | q1 |
| 67 | 4.87 | 580 | 7.484723 | q1 |
| 68 | 6.43 | 910 | 2.963885 | q1 |
| 69 | 6.24 | 910 | 5.256762 | q1 |
| 70 | 0 | 910 | 4.808346 | q1 |
| 71 | 7.68 | 910 | 5.880398 | q1 |
| 72 | 5.65 | 910 | 8.485791 | q1 |
| 73 | 1.131 | 160 | 3.706376 | FDis |
| 74 | 1.52 | 160 | 3.020752 | FDis |
| 75 | 0.97 | 160 | 3.152691 | FDis |
| 76 | 1.75 | 160 | 3.309833 | FDis |
| 77 | 0.438 | 160 | 3.973483 | FDis |
| 78 | 1.79 | 160 | 3.102858 | FDis |
| 79 | 0 | 160 | 2.963686 | FDis |
| 80 | 2.45 | 160 | 3.258303 | FDis |
| 81 | 2.65 | 250 | 3.516256 | FDis |
| 82 | 3.19 | 250 | 3.178529 | FDis |
| 83 | 3 | 250 | 2.603902 | FDis |
| 84 | 2.96 | 250 | 2.629557 | FDis |
| 85 | 2.106 | 250 | 3.305432 | FDis |
| 86 | 4.99 | 250 | 3.969306 | FDis |
| 87 | 0 | 250 | 3.211555 | FDis |
| 88 | 7.02 | 250 | 2.343015 | FDis |
| 89 | 2.14 | 285 | 2.493475 | FDis |
| 90 | 3.15 | 285 | 1.112883 | FDis |
| 91 | 4.87 | 285 | 2.145265 | FDis |
| 92 | 3.081 | 285 | 1.994107 | FDis |
| 93 | 5.3 | 285 | 2.024844 | FDis |
| 94 | 5.26 | 390 | 0.111296 | FDis |
| 95 | 2.49 | 390 | 0.147923 | FDis |
| 96 | 2.61 | 390 | 0.16836 | FDis |
| 97 | 4.75 | 390 | 0.082432 | FDis |
| 98 | 1.28 | 390 | 0.142374 | FDis |
| 99 | 1.209 | 390 | 0.039461 | FDis |
| 100 | 13.4 | 580 | 4.023276 | FDis |
| 101 | 9.36 | 580 | 2.676336 | FDis |
| 102 | 12.4 | 580 | 2.968842 | FDis |
| 103 | 4.87 | 580 | 3.318169 | FDis |
| 104 | 6.43 | 910 | 1.664361 | FDis |
| 105 | 6.24 | 910 | 3.263698 | FDis |
| 106 | 0 | 910 | 3.930695 | FDis |
| 107 | 7.68 | 910 | 2.942868 | FDis |
| 108 | 5.65 | 910 | 1.496755 | FDis |
| 109 | 1.131 | 160 | 3.798545 | CWMSLA |
| 110 | 1.52 | 160 | 0.835759 | CWMSLA |
| 111 | 0.97 | 160 | 6.319282 | CWMSLA |
| 112 | 1.75 | 160 | 6.312611 | CWMSLA |
| 113 | 0.438 | 160 | 4.969343 | CWMSLA |
| 114 | 1.79 | 160 | 6.082814 | CWMSLA |
| 115 | 0 | 160 | 6.118228 | CWMSLA |
| 116 | 2.45 | 160 | 5.052424 | CWMSLA |
| 117 | 2.65 | 250 | 6.991605 | CWMSLA |
| 118 | 3.19 | 250 | 7.775074 | CWMSLA |
| 119 | 3 | 250 | 3.945325 | CWMSLA |
| 120 | 2.96 | 250 | 4.752076 | CWMSLA |
| 121 | 2.106 | 250 | 27.58325 | CWMSLA |
| 122 | 4.99 | 250 | 4.837976 | CWMSLA |
| 123 | 0 | 250 | 4.264692 | CWMSLA |
| 124 | 7.02 | 250 | 6.650798 | CWMSLA |
| 125 | 2.14 | 285 | 19.40809 | CWMSLA |
| 126 | 3.15 | 285 | 3.983631 | CWMSLA |
| 127 | 4.87 | 285 | 9.456751 | CWMSLA |
| 128 | 3.081 | 285 | 24.07791 | CWMSLA |
| 129 | 5.3 | 285 | 9.424109 | CWMSLA |
| 130 | 5.26 | 390 | 22.76377 | CWMSLA |
| 131 | 2.49 | 390 | 30.13104 | CWMSLA |
| 132 | 2.61 | 390 | 31.83036 | CWMSLA |
| 133 | 4.75 | 390 | 26.65367 | CWMSLA |
| 134 | 1.28 | 390 | 31.53552 | CWMSLA |
| 135 | 1.209 | 390 | 19.97187 | CWMSLA |
| 136 | 13.4 | 580 | 21.98048 | CWMSLA |
| 137 | 9.36 | 580 | 20.0279 | CWMSLA |
| 138 | 12.4 | 580 | 19.05198 | CWMSLA |
| 139 | 4.87 | 580 | 13.34766 | CWMSLA |
| 140 | 6.43 | 910 | 13.07541 | CWMSLA |
| 141 | 6.24 | 910 | 8.295613 | CWMSLA |
| 142 | 0 | 910 | 17.1244 | CWMSLA |
| 143 | 7.68 | 910 | 7.275746 | CWMSLA |
| 144 | 5.65 | 910 | 37.26232 | CWMSLA |
| 145 | 1.131 | 160 | 19.00648 | CWMLDMC |
| 146 | 1.52 | 160 | 13.61622 | CWMLDMC |
| 147 | 0.97 | 160 | 41.91488 | CWMLDMC |
| 148 | 1.75 | 160 | 17.45631 | CWMLDMC |
| 149 | 0.438 | 160 | 18.19874 | CWMLDMC |
| 150 | 1.79 | 160 | 38.78612 | CWMLDMC |
| 151 | 0 | 160 | 34.70401 | CWMLDMC |
| 152 | 2.45 | 160 | 22.59705 | CWMLDMC |
| 153 | 2.65 | 250 | 39.7971 | CWMLDMC |
| 154 | 3.19 | 250 | 37.01491 | CWMLDMC |
| 155 | 3 | 250 | 26.24408 | CWMLDMC |
| 156 | 2.96 | 250 | 30.73069 | CWMLDMC |
| 157 | 2.106 | 250 | 33.04391 | CWMLDMC |
| 158 | 4.99 | 250 | 27.89078 | CWMLDMC |
| 159 | 0 | 250 | 31.88888 | CWMLDMC |
| 160 | 7.02 | 250 | 36.18298 | CWMLDMC |
| 161 | 2.14 | 285 | 26.91754 | CWMLDMC |
| 162 | 3.15 | 285 | 31.5725 | CWMLDMC |
| 163 | 4.87 | 285 | 39.20079 | CWMLDMC |
| 164 | 3.081 | 285 | 31.70071 | CWMLDMC |
| 165 | 5.3 | 285 | 24.37365 | CWMLDMC |
| 166 | 5.26 | 390 | 42.10611 | CWMLDMC |
| 167 | 2.49 | 390 | 4.024983 | CWMLDMC |
| 168 | 2.61 | 390 | 4.063886 | CWMLDMC |
| 169 | 4.75 | 390 | 5.389142 | CWMLDMC |
| 170 | 1.28 | 390 | 9.579278 | CWMLDMC |
| 171 | 1.209 | 390 | 3.260251 | CWMLDMC |
| 172 | 13.4 | 580 | 36.237 | CWMLDMC |
| 173 | 9.36 | 580 | 33.955 | CWMLDMC |
| 174 | 12.4 | 580 | 25.322 | CWMLDMC |
| 175 | 4.87 | 580 | 36.264 | CWMLDMC |
| 176 | 6.43 | 910 | 36.75512 | CWMLDMC |
| 177 | 6.24 | 910 | 38.76778 | CWMLDMC |
| 178 | 0 | 910 | 20.39792 | CWMLDMC |
| 179 | 7.68 | 910 | 25.5847 | CWMLDMC |
| 180 | 5.65 | 910 | 27.00773 | CWMLDMC |
| 181 | 1.131 | 160 | 18.66667 | CWMheight |
| 182 | 1.52 | 160 | 35.25 | CWMheight |
| 183 | 0.97 | 160 | 17.09091 | CWMheight |
| 184 | 1.75 | 160 | 27.04545 | CWMheight |
| 185 | 0.438 | 160 | 26.67568 | CWMheight |
| 186 | 1.79 | 160 | 18.66667 | CWMheight |
| 187 | 0 | 160 | 35.25 | CWMheight |
| 188 | 2.45 | 160 | 17.09091 | CWMheight |
| 189 | 2.65 | 250 | 20.03125 | CWMheight |
| 190 | 3.19 | 250 | 14.33929 | CWMheight |
| 191 | 3 | 250 | 11.37838 | CWMheight |
| 192 | 2.96 | 250 | 5.735616 | CWMheight |
| 193 | 2.106 | 250 | 9.231132 | CWMheight |
| 194 | 4.99 | 250 | 24.57895 | CWMheight |
| 195 | 0 | 250 | 10.65306 | CWMheight |
| 196 | 7.02 | 250 | 14.62069 | CWMheight |
| 197 | 2.14 | 285 | 23.54182 | CWMheight |
| 198 | 3.15 | 285 | 6.06383 | CWMheight |
| 199 | 4.87 | 285 | 35.11111 | CWMheight |
| 200 | 3.081 | 285 | 32.27119 | CWMheight |
| 201 | 5.3 | 285 | 57.048 | CWMheight |
| 202 | 5.26 | 390 | 8.902439 | CWMheight |
| 203 | 2.49 | 390 | 11.73684 | CWMheight |
| 204 | 2.61 | 390 | 9.538462 | CWMheight |
| 205 | 4.75 | 390 | 8.307692 | CWMheight |
| 206 | 1.28 | 390 | 20.27273 | CWMheight |
| 207 | 1.209 | 390 | 6.902439 | CWMheight |
| 208 | 13.4 | 580 | 14.98305 | CWMheight |
| 209 | 9.36 | 580 | 6.45 | CWMheight |
| 210 | 12.4 | 580 | 12.02222 | CWMheight |
| 211 | 4.87 | 580 | 13.95 | CWMheight |
| 212 | 6.43 | 910 | 38.21429 | CWMheight |
| 213 | 6.24 | 910 | 36.46667 | CWMheight |
| 214 | 0 | 910 | 36.5 | CWMheight |
| 215 | 7.68 | 910 | 24.27273 | CWMheight |
| 216 | 5.65 | 910 | 21.66667 | CWMheight |

**Appendix S6**. Raw data used to picture variations in taxonomic (q0, q1) and functional (FDis, CWMSLA, CWMLDMC, CWMheight) diversity across precipitation and phosphorus (P) gradients in surrounding rangelands.

| number | P | precipitation | Plant biodiversity | Class |
| --- | --- | --- | --- | --- |
| 1 | 10 | 160 | 7 | q0 |
| 2 | 11 | 160 | 7 | q0 |
| 3 | 15 | 160 | 10 | q0 |
| 4 | 11 | 160 | 13 | q0 |
| 5 | 6 | 160 | 19 | q0 |
| 6 | 7 | 250 | 17 | q0 |
| 7 | 0 | 250 | 10 | q0 |
| 8 | 11 | 250 | 12 | q0 |
| 9 | 8 | 250 | 10 | q0 |
| 10 | 5 | 250 | 1 | q0 |
| 11 | 5 | 285 | 10 | q0 |
| 12 | 5 | 285 | 15 | q0 |
| 13 | 5 | 285 | 12 | q0 |
| 14 | 12 | 285 | 19 | q0 |
| 15 | 9 | 285 | 28 | q0 |
| 16 | 13 | 285 | 18 | q0 |
| 17 | 11 | 390 | 8 | q0 |
| 18 | 9 | 390 | 12 | q0 |
| 19 | 8 | 390 | 8 | q0 |
| 20 | 8 | 390 | 7 | q0 |
| 21 | 9 | 580 | 5 | q0 |
| 22 | 16 | 580 | 10 | q0 |
| 23 | 22 | 580 | 11 | q0 |
| 24 | 6 | 580 | 9 | q0 |
| 25 | 11 | 580 | 7 | q0 |
| 26 | 4 | 910 | 9 | q0 |
| 27 | 4 | 910 | 9 | q0 |
| 28 | 5 | 910 | 10 | q0 |
| 29 | 5 | 910 | 10 | q0 |
| 30 | 10 | 160 | 3.50336 | q1 |
| 31 | 11 | 160 | 4.843777 | q1 |
| 32 | 15 | 160 | 7.368652 | q1 |
| 33 | 11 | 160 | 8.552141 | q1 |
| 34 | 6 | 160 | 12.01534 | q1 |
| 35 | 7 | 250 | 8.33345 | q1 |
| 36 | 0 | 250 | 6.459877 | q1 |
| 37 | 11 | 250 | 7.024361 | q1 |
| 38 | 8 | 250 | 9.126316 | q1 |
| 39 | 5 | 250 | 1 | q1 |
| 40 | 5 | 285 | 6.262122 | q1 |
| 41 | 5 | 285 | 7.075421 | q1 |
| 42 | 5 | 285 | 5.106353 | q1 |
| 43 | 12 | 285 | 14.36992 | q1 |
| 44 | 9 | 285 | 19.18875 | q1 |
| 45 | 13 | 285 | 8.499446 | q1 |
| 46 | 11 | 390 | 5.468653 | q1 |
| 47 | 9 | 390 | 3.825557 | q1 |
| 48 | 8 | 390 | 6.649281 | q1 |
| 49 | 8 | 390 | 6.008759 | q1 |
| 50 | 9 | 580 | 4.757043 | q1 |
| 51 | 16 | 580 | 4.008837 | q1 |
| 52 | 22 | 580 | 5.783413 | q1 |
| 53 | 6 | 580 | 3.616579 | q1 |
| 54 | 11 | 580 | 5.625415 | q1 |
| 55 | 4 | 910 | 5.642031 | q1 |
| 56 | 4 | 910 | 7.22556 | q1 |
| 57 | 5 | 910 | 3.875216 | q1 |
| 58 | 5 | 910 | 5.407304 | q1 |
| 59 | 13 | 160 | 5 | q0 |
| 60 | 17 | 160 | 9 | q0 |
| 61 | 13 | 160 | 6 | q0 |
| 62 | 13 | 160 | 1 | q0 |
| 63 | 9 | 160 | 10 | q0 |
| 64 | 12 | 160 | 12 | q0 |
| 65 | 0 | 160 | 8 | q0 |
| 66 | 5 | 160 | 7 | q0 |
| 67 | 12 | 250 | 10 | q0 |
| 68 | 6 | 250 | 13 | q0 |
| 69 | 8 | 250 | 16 | q0 |
| 70 | 10 | 250 | 12 | q0 |
| 71 | 15 | 250 | 17 | q0 |
| 72 | 4 | 250 | 12 | q0 |
| 73 | 0 | 250 | 17 | q0 |
| 74 | 8 | 250 | 19 | q0 |
| 75 | 4 | 285 | 12 | q0 |
| 76 | 5 | 285 | 5 | q0 |
| 77 | 6 | 285 | 5 | q0 |
| 78 | 5 | 285 | 6 | q0 |
| 79 | 5 | 285 | 6 | q0 |
| 80 | 6 | 390 | 6 | q0 |
| 81 | 17 | 390 | 6 | q0 |
| 82 | 25 | 390 | 3 | q0 |
| 83 | 5 | 390 | 2 | q0 |
| 84 | 12 | 390 | 3 | q0 |
| 85 | 15 | 390 | 6 | q0 |
| 86 | 4 | 580 | 10 | q0 |
| 87 | 6 | 580 | 5 | q0 |
| 88 | 5 | 580 | 10 | q0 |
| 89 | 8 | 580 | 9 | q0 |
| 90 | 5 | 910 | 10 | q0 |
| 91 | 5 | 910 | 8 | q0 |
| 92 | 0 | 910 | 7 | q0 |
| 93 | 11 | 910 | 7 | q0 |
| 94 | 13 | 910 | 9 | q0 |
| 95 | 13 | 160 | 5 | q0 |
| 96 | 17 | 160 | 5.107462 | q1 |
| 97 | 13 | 160 | 3.69149 | q1 |
| 98 | 13 | 160 | 1 | q1 |
| 99 | 9 | 160 | 6.705189 | q1 |
| 100 | 12 | 160 | 8.184661 | q1 |
| 101 | 0 | 160 | 5.218197 | q1 |
| 102 | 5 | 160 | 4.850848 | q1 |
| 103 | 12 | 250 | 7.562765 | q1 |
| 104 | 6 | 250 | 6.389848 | q1 |
| 105 | 8 | 250 | 8.875432 | q1 |
| 106 | 10 | 250 | 10.35417 | q1 |
| 107 | 15 | 250 | 10.84946 | q1 |
| 108 | 4 | 250 | 8.95776 | q1 |
| 109 | 0 | 250 | 5.267109 | q1 |
| 110 | 8 | 250 | 10.29991 | q1 |
| 111 | 4 | 285 | 7.124047 | q1 |
| 112 | 5 | 285 | 2.522911 | q1 |
| 113 | 6 | 285 | 2.641892 | q1 |
| 114 | 5 | 285 | 2.845204 | q1 |
| 115 | 5 | 285 | 3.660564 | q1 |
| 116 | 6 | 390 | 3.303058 | q1 |
| 117 | 17 | 390 | 4.348513 | q1 |
| 118 | 25 | 390 | 2.363525 | q1 |
| 119 | 5 | 390 | 1.417714 | q1 |
| 120 | 12 | 390 | 2.12382 | q1 |
| 121 | 15 | 390 | 3.173848 | q1 |
| 122 | 4 | 580 | 6.433076 | q1 |
| 123 | 6 | 580 | 3.793634 | q1 |
| 124 | 5 | 580 | 6.656927 | q1 |
| 125 | 8 | 580 | 7.484723 | q1 |
| 126 | 5 | 910 | 2.963885 | q1 |
| 127 | 5 | 910 | 5.256762 | q1 |
| 128 | 0 | 910 | 4.808346 | q1 |
| 129 | 11 | 910 | 5.880398 | q1 |
| 130 | 13 | 910 | 8.485791 | q1 |
| 132 | 10 | 160 | 0.180372 | FDis |
| 133 | 11 | 160 | 0.155588 | FDis |
| 134 | 15 | 160 | 0.201665 | FDis |
| 135 | 11 | 160 | 0.225079 | FDis |
| 136 | 6 | 160 | 0.086298 | FDis |
| 137 | 7 | 250 | 3.379228 | FDis |
| 138 | 0 | 250 | 3.099583 | FDis |
| 139 | 11 | 250 | 3.529794 | FDis |
| 140 | 8 | 250 | 4.42575 | FDis |
| 141 | 5 | 250 | 3.566106 | FDis |
| 142 | 5 | 285 | 3.12324 | FDis |
| 143 | 5 | 285 | 3.101267 | FDis |
| 144 | 5 | 285 | 2.275625 | FDis |
| 145 | 12 | 285 | 2.863935 | FDis |
| 146 | 9 | 285 | 3.473892 | FDis |
| 147 | 13 | 285 | 0.91311 | FDis |
| 148 | 11 | 390 | 3.496398 | FDis |
| 149 | 9 | 390 | 3.671443 | FDis |
| 150 | 8 | 390 | 2.876939 | FDis |
| 151 | 8 | 390 | 3.827532 | FDis |
| 152 | 9 | 580 | 4.217153 | FDis |
| 153 | 16 | 580 | 3.072985 | FDis |
| 154 | 22 | 580 | 2.775984 | FDis |
| 155 | 6 | 580 | 3.25291 | FDis |
| 156 | 11 | 580 | 3.674472 | FDis |
| 157 | 4 | 910 | 2.736283 | FDis |
| 158 | 4 | 910 | 3.096878 | FDis |
| 159 | 5 | 910 | 2.000704 | FDis |
| 160 | 5 | 910 | 2.991984 | FDis |
| 161 | 10 | 160 | 11.09963 | CWMSLA |
| 162 | 11 | 160 | 9.323366 | CWMSLA |
| 163 | 15 | 160 | 9.323366 | CWMSLA |
| 164 | 11 | 160 | 9.661976 | CWMSLA |
| 165 | 6 | 160 | 14.715 | CWMSLA |
| 166 | 7 | 250 | 20.72086 | CWMSLA |
| 167 | 0 | 250 | 8.842388 | CWMSLA |
| 168 | 11 | 250 | 25.56951 | CWMSLA |
| 169 | 8 | 250 | 18.57794 | CWMSLA |
| 170 | 5 | 250 | 11.73916 | CWMSLA |
| 171 | 5 | 285 | 21.08656 | CWMSLA |
| 172 | 5 | 285 | 18.94889 | CWMSLA |
| 173 | 5 | 285 | 9.239883 | CWMSLA |
| 174 | 12 | 285 | 18.52119 | CWMSLA |
| 175 | 9 | 285 | 17.31932 | CWMSLA |
| 176 | 13 | 285 | 18.88097 | CWMSLA |
| 177 | 11 | 390 | 3.685299 | CWMSLA |
| 178 | 9 | 390 | 5.73429 | CWMSLA |
| 179 | 8 | 390 | 5.025427 | CWMSLA |
| 180 | 8 | 390 | 3.107565 | CWMSLA |
| 181 | 9 | 580 | 23.426 | CWMSLA |
| 182 | 16 | 580 | 15.826 | CWMSLA |
| 183 | 22 | 580 | 10.625 | CWMSLA |
| 184 | 6 | 580 | 22.755 | CWMSLA |
| 185 | 11 | 580 | 29.376 | CWMSLA |
| 186 | 4 | 910 | 34.31404 | CWMSLA |
| 187 | 4 | 910 | 36.61569 | CWMSLA |
| 188 | 5 | 910 | 33.86564 | CWMSLA |
| 189 | 5 | 910 | 16.26584 | CWMSLA |
| 190 | 10 | 160 | 38.40789 | CWMLDMC |
| 191 | 11 | 160 | 38.59672 | CWMLDMC |
| 192 | 15 | 160 | 41.99198 | CWMLDMC |
| 193 | 11 | 160 | 34.24188 | CWMLDMC |
| 194 | 6 | 160 | 31.05542 | CWMLDMC |
| 195 | 7 | 250 | 23.58477 | CWMLDMC |
| 196 | 0 | 250 | 16.99264 | CWMLDMC |
| 197 | 11 | 250 | 21.41133 | CWMLDMC |
| 198 | 8 | 250 | 40.39972 | CWMLDMC |
| 199 | 5 | 250 | 42.9295 | CWMLDMC |
| 200 | 5 | 285 | 21.08656 | CWMLDMC |
| 201 | 5 | 285 | 18.94889 | CWMLDMC |
| 202 | 5 | 285 | 9.239883 | CWMLDMC |
| 203 | 12 | 285 | 18.52119 | CWMLDMC |
| 204 | 9 | 285 | 27.31932 | CWMLDMC |
| 205 | 13 | 285 | 28.88097 | CWMLDMC |
| 206 | 11 | 390 | 31.08403 | CWMLDMC |
| 207 | 9 | 390 | 35.48502 | CWMLDMC |
| 208 | 8 | 390 | 57.14048 | CWMLDMC |
| 209 | 8 | 390 | 43.84216 | CWMLDMC |
| 210 | 9 | 580 | 37.37383 | CWMLDMC |
| 211 | 16 | 580 | 16.14862 | CWMLDMC |
| 212 | 22 | 580 | 31.7994 | CWMLDMC |
| 213 | 6 | 580 | 17.45539 | CWMLDMC |
| 214 | 11 | 580 | 21.80595 | CWMLDMC |
| 215 | 4 | 910 | 33.45375 | CWMLDMC |
| 216 | 4 | 910 | 20.53114 | CWMLDMC |
| 217 | 5 | 910 | 25.93233 | CWMLDMC |
| 218 | 5 | 910 | 27.03477 | CWMLDMC |
| 219 | 10 | 160 | 21.17823 | CWMheight |
| 220 | 11 | 160 | 17.02537 | CWMheight |
| 221 | 15 | 160 | 16.01193 | CWMheight |
| 222 | 11 | 160 | 16.62698 | CWMheight |
| 223 | 6 | 160 | 8.907 | CWMheight |
| 224 | 7 | 250 | 24.56226 | CWMheight |
| 225 | 0 | 250 | 22.86395 | CWMheight |
| 226 | 11 | 250 | 22.81667 | CWMheight |
| 227 | 8 | 250 | 23.95806 | CWMheight |
| 228 | 5 | 250 | 26.18 | CWMheight |
| 229 | 5 | 285 | 21.08656 | CWMheight |
| 230 | 5 | 285 | 18.94889 | CWMheight |
| 231 | 5 | 285 | 9.239883 | CWMheight |
| 232 | 12 | 285 | 18.52119 | CWMheight |
| 233 | 9 | 285 | 27.31932 | CWMheight |
| 234 | 13 | 285 | 28.88097 | CWMheight |
| 235 | 11 | 390 | 12.28 | CWMheight |
| 236 | 9 | 390 | 7.311111 | CWMheight |
| 237 | 8 | 390 | 11.85714 | CWMheight |
| 238 | 8 | 390 | 13.52941 | CWMheight |
| 239 | 9 | 580 | 6.99005 | CWMheight |
| 240 | 16 | 580 | 10.78409 | CWMheight |
| 241 | 22 | 580 | 8.982063 | CWMheight |
| 242 | 6 | 580 | 17.84906 | CWMheight |
| 243 | 11 | 580 | 53.125 | CWMheight |
| 244 | 4 | 910 | 13.81818 | CWMheight |
| 245 | 4 | 910 | 35.69697 | CWMheight |
| 246 | 5 | 910 | 34.71287 | CWMheight |
| 247 | 5 | 910 | 31.7037 | CWMheight |
